# Modulating Activity in the Prefrontal Cortex Changes Intertemporal Choice for Loss: A Transcranial Direct Current Stimulation Study

**DOI:** 10.3389/fnhum.2019.00167

**Published:** 2019-05-24

**Authors:** Guanxing Xiong, Xi Li, Zhiqiang Dong, Shenggang Cai, Jianye Huang, Qian Li

**Affiliations:** ^1^School of Economics and Management, South China Normal University, Guangzhou, China; ^2^Key Lab for Behavioral Economic Science & Technology, South China Normal University, Guangzhou, China

**Keywords:** delay discounting, intertemporal choice, loss, dorsolateral prefrontal cortex, transcranial direct current stimulation

## Abstract

Intertemporal choice refers to decisions involving tradeoffs between costs and benefits occurring at different times. Studies have found that weighting the time and benefits during decision-making involves a complex neural network that includes the dorsolateral prefrontal cortex (DLPFC). However, in contrast to literature regarding intertemporal choice for gains, studies have not provided causal evidence that the DLPFC is involved in intertemporal choice for losses. We examined whether bifrontal transcranial direct current stimulation (tDCS) applied over the right and left prefrontal cortex can alter the balance of intertemporal preference in the loss condition. A total of 60 participants performed delay discounting tasks for losses while receiving either right anodal/left cathodal, left anodal/right cathodal, or sham stimulation. The results showed that participants tended to choose larger delayed losses after receiving left anodal/right cathodal tDCS. Left anodal/right cathodal tDCS significantly decreased the discounting rate compared with the sham stimulation. These findings confirm that DLPFC activity is critical during intertemporal decision-making for losses.

## Introduction

In their daily lives, people must make decisions regarding tradeoffs between benefits and costs that occur at various times. For example, people may be faced with the choice of whether quitting smoking; this decision contains two options—the first option is the short-term pain of quitting cigarette smoking recently, whereas the second option is the long-term pain of falling ill (even suffering lung cancer) in the future. Scholars deem this type of decision as intertemporal choice, which refers to a human beings’ tendency to discount future benefits/costs while facing decisions that involve a smaller immediate gain/loss and a larger future one (Ainslie, 2001). From an economics perspective, this preference can be understood in terms of delay discounting (Frederick et al., 2002). Economists and psychologists sometimes use delay discounting as a tool to assess individuals with various forms of addiction and gambling habits (Hecht et al., 2013).

Brain imaging studies have indicated that intertemporal choice for gains is associated with activities in the dorsolateral prefrontal cortex (DLPFC). Peters and Büchel (2011) indicated that the primary region facilitating the cognitive control process involved in delay discounting is the DLPFC. Li et al. (2010) showed that the resting-state functional connectivity between the ventromedial prefrontal cortex and DLPFC is negatively related to delay discounting rates. In addition to the neuroimaging studies, brain stimulation technologies were also used to explore the neural basis of intertemporal choice for gains. Hecht et al. (2013) found that, compared with a sham stimulation, participants receiving bilateral DLPFC stimulation exhibited a greater preference for smaller immediate gains over larger delayed ones. He et al. (2016) showed that brain stimulation over the left DLPFC decreased the delay discounting rate in intertemporal choice.

Neuroimaging studies have primarily focused on intertemporal choice for gain, and few studies have investigated intertemporal choice for loss. Therefore, the neural mechanism that causes discounting future losses remains unidentified. Discounting may have a different neural mechanism for future losses and gains. The sign effect, proposed by behavioral researchers, describes the phenomenon wherein individual discounts delay losses less steeply than they do gains (Loewenstein, 1987). Gain–loss asymmetry in delay discounting was first proposed by Loewenstein and Prelec (1992) and has since been explored by several studies (Caplin and Leahy, 2001; Zhang et al., 2016). In a functional magnetic resonance imaging (fMRI) study, Xu et al. (2009) supported the asymmetric hypothesis and described the asymmetric activity pattern underlying the process of discounting future gains and losses. The authors investigated the asymmetric mechanisms of gain- and loss-associated delay discounting, with greater activation occurring mainly in the DLPFC and other encephalic regions during the discounting task of loss.

The present study aims to fill the gap in the literature by examining the causal role of the DLPFC for intertemporal choice for losses. Neuroimaging studies have demonstrated that decision-related activation is observed in the DLPFC for delay discounting tasks with loss (Xu et al., 2009). Moreover, intertemporal choice is closely related to individuals’ cognitive control and impulsivity (Takahashi, 2005; Wittmann and Paulus, 2008). Neural studies have indicated that DLPFC is the main region involved in the process of cognitive control and impulsivity (Figner et al., 2010; Shen et al., 2016). Furthermore, Zhang et al. (2016) suggested that the valuation of loss outcome may activate negative emotion. fMRI and transcranial direct current stimulation (tDCS) studies have demonstrated that DLPFC and the amygdala (the emotion-related encephalic region) are involved in brain function connectivity (Bishop, 2009; Ironside et al., 2016). Pan et al. (2019) proposed that decreased activation of the rDLPFC specifically decreased subjective probability of rejections under incredible threat. Therefore, by exploring the causal relationship between brain area and intertemporal choice with loss, we believe that DLPFC would be an appropriate choice.

Noninvasive brain stimulation instruments have been used in the studies of decision-making to explore the causal relationship between the encephalic region and the relative behavior. For example, Sheffer et al. (2013) demonstrated that increasing activity in the left DLPFC with high-frequency repetitive transcranial magnetic stimulation (HF rTMS) would decrease impulsive decision-making. Theta burst stimulation (TBS) and tDCS were also often used in neural stimulation experiments related to decision-making (Cho et al., 2010; Brevet-Aeby et al., 2016; He et al., 2016; Zack et al., 2016). Because tDCS is a safer technology and has been widely used in research on brain stimulation in healthy groups, we adopt the tDCS as our simulation method in this study. tDCS is a neuromodulation technique that is capable of inducing sensory, motor, perceptual, and cognitive effects in healthy participants (Utz et al., 2010). Studies on the physiological effects of tDCS have revealed that tDCS does indeed modulate synaptic strength within the cortex (Stagg and Nitsche, 2011). The dual polarity of tDCS enables the exploration of the accurate effect of prefrontal activity on the cortex; it can activate one side of the brain region through the anodal electrode and simultaneously suppress the other side of the brain region through the cathodal electrode. Using a bifrontal montage, we were able to *upregulate* activity in one hemisphere and *downregulate* it in the contralateral hemisphere.

By modulating activity in the DLPFC, the current study explored changes in an individual’s delay discounting attitude of loss before and after tDCS. In particular, we hypothesized that tDCS of the DLPFC influences participants’ choice of present or distant future monetary losses, specifically left anodal/right cathodal tDCS stimulation leads participants to choose larger delayed losses than sham stimulation does. The capacity of tDCS to influence human behavior is currently being questioned. Hence, to determine which tDCS protocols are effective, the implementation of methodologically sound studies is crucial for the brain stimulation literature. Therefore, the present study is timely and important.

## Materials and Methods

### Experimental Design

Our experiment had a single-blind design. The stimulation type (RH anodal/LH cathodal vs. RH cathodal/LH anodal vs. sham) was manipulated between subjects, and the trials of the intertemporal choice task were manipulated within subjects, resulting in a mixed experimental design. In specific, participants randomly underwent one of the three stimulation conditions: (1) active stimulation with the anodal electrode placed over the right DLPFC and the cathodal electrode over the left DLPFC (referred to as “RH anodal/LH cathodal condition”); (2) active stimulation with the anodal electrode over the left DLPFC and the cathodal electrode over the right DLPFC (referred to as “LH anodal/RH cathodal condition”); and (3) sham stimulation (referred to as “control condition”), in which participants were initially stimulated for only 30 s in order to let them feel the tingling sensation associated with tDCS, and then the stimulation faded gradually without the participants’ awareness.

### Subjects

We recruited 60 healthy college students (32 females; mean age 20.26 years, ranging from 18 to 24) to participate in our experiment. All the participants were healthy, were right-handed, and had normal or corrected-to-normal vision. They were naïve to tDCS and delay discounting tasks, and had no history of psychiatric illness or neurological disorders. The participants were randomly assigned to receive right anodal/left cathodal tDCS (*n* = 20, 11 female participants), left anodal/right cathodal tDCS (*n* = 20, 10 female participants), or sham stimulation (*n* = 20, 11 female participants). The final payoff was a fixed show-up fee of RMB 50 (approximately US$8). The participants were provided with informed written consent before entering the study, which was approved by the ethics committee of South China Normal University. The safety procedures were followed in accordance with Non-invasive Brain Stimulation indications (Poreisz et al., 2007).

### Transcranial Direct Current Stimulation

This study employed tDCS, which is a simple, painless, and noninvasive technique for modulating brain activity that applies a low-intensity direct current. The current was delivered through battery-driven constant stimulation (DC-STIMULATOR; NeuroConn, Ilmenau, Germany) using two saline-soaked surface sponge electrodes (5 cm × 7 cm). The anodal stimulation increases cortical excitability, whereas cathodal stimulation decreases it (Nitsche et al., 2003).

For the RH anodal/LH cathodal stimulation, the anode was placed over the F4 [according to the international electroencephalography (EEG) 10/20 system] and the cathode over the F3. For the LH anodal/RH cathodal stimulation, the anode was placed over the F3 and the cathode was placed over the F4. For the participants under the sham stimulation condition, half of the electrodes were placed at the same position as that of the RH anodal/LH cathodal stimulation, and the other half of the electrodes were placed at the same position as that of the LH anodal/RH cathodal stimulation; however, the stimulator was only switched on for the initial 30 s. This method of sham stimulation has been shown to be reliable (Gandiga et al., 2006).

**Figure 1:**
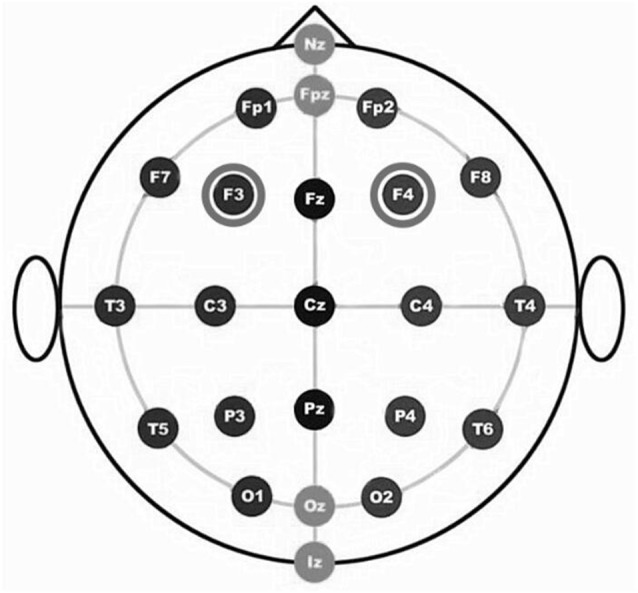
The figure is based on International EEG 10-20 system. Schematic drawing of electrode positions for transcranial direct current stimulation (tDCS) of the dorsolateral prefrontal cortex (DLPFC).

The constant current of 2 mA was applied with 30 s of ramping up and down; the safety and efficiency of this approach has been demonstrated in previous studies. After 8 min of stimulation, the participants were asked to complete the second task with the stimulation continually being delivered until they finished the whole experiment. We adopted a bifrontal electrode montage to enhance the activity of one side of the DLPFC and simultaneously diminishing the other side (Figure 1).

### Task and Procedure

The experiment was based on a delay discounting task (Wang and Dvorak, 2010) that measures a participant’s intertemporal preference. Future discounting was assessed using 24 choice options. In each choice, the participants were presented with two monetary options: a specified amount tomorrow or a larger amount (24 choices, ranging from RMB 84 to RMB 1,200) after a specified delay (2–341 days). Before the tDCS stimulation, 12 of the choices were presented, and the other 12 choices were presented after the tDCS stimulation. We asked participants questions, such as, “Would you prefer to lose 120 Yuan tomorrow or 450 Yuan in 31 days?” The difference between a smaller, sooner (SS) loss and a larger, later (LL) loss indicates the following hyperbolic discount parameter *k* (Kirby and Santiesteban, 2003):

k=(future$−now$)/(delay(indays)×now$)

**Figure 2:**
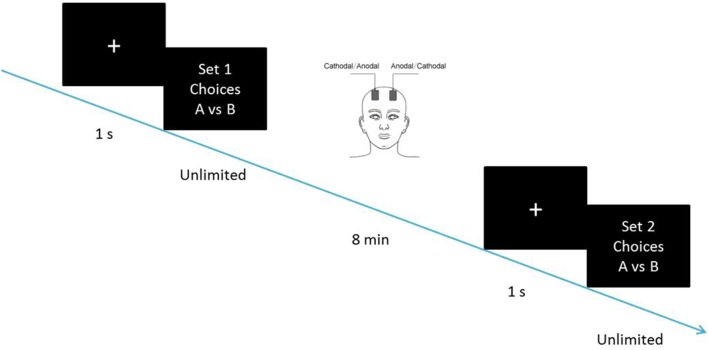
Schematic representation of the experimental design. After 8 min of stimulation, each participant was asked to complete the second task, with the stimulation being continually delivered.

If the SS is for now without delay. Choices over such a range reveal where one begins to prefer to lose larger, later money; Individual discounting parameters were computed as the geometric mean of the *k*-values bounding this preference switch (Kirby and Maraković, 1996). Their responses were used to compute the discounting parameters before and after the tDCS stimulation, as the dependent measure. The participants were required to complete the first set of choices before receiving tDCS. After 8 min of stimulation, they were required to complete the second set of choices, with the stimulation being delivered continually (Figure 2). The two sets of choices were homogeneous tasks, in which the hyperbolic discounting rates of choice had identical content such as delay discounting rates of 0.000490998, 0.000974659 and 0.00195122. The choices were mixed up in random order before being presented to the participants.

## Results

**Figure 3:**
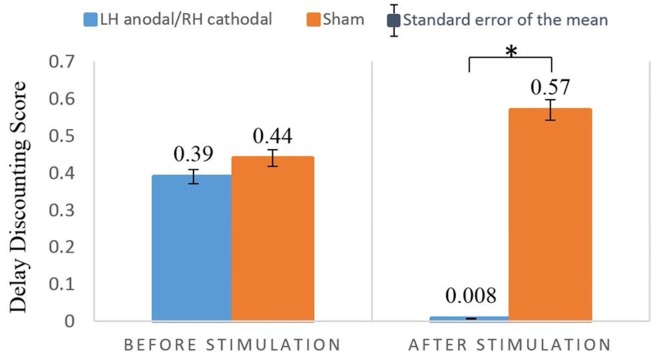
Delay discounting before and after stimulation across treatments. After LH anodal/RH cathodal tDCS, the participants’ delay discounting scores were significantly lower. *p < 0.05.

One female participant (in the sham stimulation group) was excessively nervous, and therefore did not complete the entire experiment. Hence, her data was excluded from the analysis. Because the main purpose of our study was to investigate differences between the treatment group and the sham group, we employed two sets of 2 × 2 analysis of variance (ANOVA) analyses. SPSS (version 21) was used in our analyses. First, we performed a two-way ANOVA with the treatment (LH anodal/RH cathodal and sham) as a between-subjects factor and turn (before/after tDCS) as a within-subjects factor. The 2 (treatment) × 2 (turn) mixed-model ANOVA revealed that the main effect for treatment was nonsignificant (*F*_(1,37)_ = 2.50, *p* = 0.12, *η*^2^ = 0.06), and the main effect for turn was also nonsignificant (*F*_(1,37)_ = 1.13, *p* = 0.29, *η*^2^ = 0.03). However, the parameter of the treatment by turn interaction was significant (*F*_(1,37)_ = 4.85, *p* = 0.034, *η*^2^ = 0.12). The *post hoc* analysis showed that before the stimulation, no significant differences were found between the LH anodal/RH cathodal group and sham group (*F*_(1,37)_ = 0.04, *p* = 0.84, *η*^2^ = 0.001), which implied that the participants’ degree of delay discounting did not differ across treatments. However, after LH anodal/RH cathodal tDCS, the participants’ average score of delay discounting was significantly lower than that of the sham group (*F*_(1,37)_ = 7.93, *p* = 0.008, *η*^2^ = 0.18). Moreover, because the two sets of choices were homogeneous tasks, the two scores of discount rates in the LH anodal/RH cathodal group were significantly different (*F*_(1,37)_ = 7.93, *p* = 0.025, *η*^2^ = 0.13). No difference was noted in the delay discounting before and after stimulation in the sham group (*F*_(1,37)_ = 0.63, *p* = 0.432, *η*^2^ = 0.02). We can conclude that the LH anodal/RH cathodal tDCS stimulation significantly changed the participants’ intertemporal choice for losses. The detailed information is shown in Figure 3.

**Figure 4:**
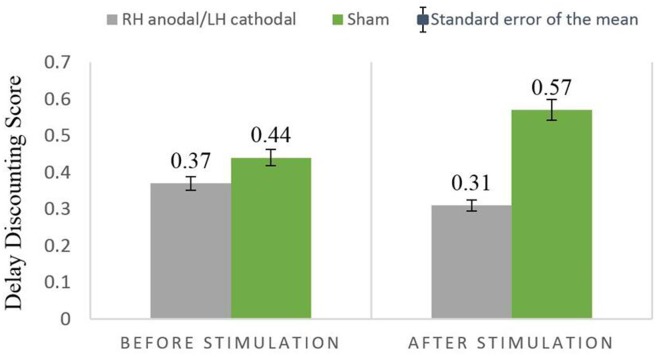
Delay discounting before and after stimulation across treatments. The delay discounting scores between the RH anodal/LH cathoal and sham groups had no significant difference.

We then performed a two-way ANOVA with treatment (RH anodal/LH cathodal and sham) as a between-subjects factor and turn (before/after tDCS) as a within-subjects factor. The main effects of treatment (*F*_(1,37)_ = 0.58, *p* = 0.45, *η*^2^ = 0.015) and turn (*F*_(1,37)_ = 0.1, *p* = 0.75, *η*^2^ = 0.003) were not observed. The parameter of treatment by turn interaction was nonsignificant (*F*_(1,37)_ = 0.63, *p* = 0.43, *η*^2^ = 0.017). RH anodal/LH cathodal tDCS showed no significant difference when compared with the sham stimulation (Figure 4).

## Discussion

The results revealed that left anodal/right cathodal tDCS significantly decreased the discounting rate compared with the sham stimulation. The participants tended to choose larger delayed losses in the left anodal/right cathodal group. However, no differences were exhibited between the group for right anodal/left cathodal tDCS and the sham stimulation group. Right anodal/left cathodal tDCS stimulation triggered no causal effects on people’s intertemporal choices regarding losses. The current study and numerous tDCS studies from the field of self-control and aggression (Kelley et al., 2013; Riva et al., 2014; Pripfl and Lamm, 2015; Carter et al., 2018) demonstrate the potential of this tool in affecting human behavior.

Several fMRI studies have linked the left DLPFC to delay discounting in intertemporal choice (Weber and Huettel, 2008; Xu et al., 2009; Liu et al., 2012). Moreover, other brain stimulation studies have revealed that stimulating DLPFC-affected participants’ intertemporal choice for gains led them to opt for increasingly immediate gratification (Hecht et al., 2013; He et al., 2016). However, these studies have not provided a direct causal link between brain structures and the behaviors of discounting future losses. The primary finding of the current study is that the modulation of activity in the DLPFC (LH anodal/RH cathodal) using tDCS can influence intertemporal decision-making for loss in healthy individuals, thus showing that LH anodal/RH cathodal stimulation appears to have a stronger effect on behavioral changes than the sham stimulation does. The study results enhanced understanding of the causal relationship between brain structure and delay discounting for intertemporal loss.

From an economics perspective, the value of a delayed outcome is discounted. Rational people should postpone the negative experience of losing money because future disutility will be discounted, and therefore, is lower than the current disutility (Loewenstein and Prelec, 1992; Harvey, 1994). However, several studies have shown that individuals often exhibit the opposite pattern. For example, Loewenstein (1987) indicated that participants are willing to pay increased amounts to avoid the electric shock in short-term delays than in long-term delays; Harris (2012) also reported some similar conclusions. We suggest that determining whether hastening the negative experience is good or not depends on the nature of the negative events. For the negative events of losing money, postponement notifies the minimization of the costs of economic rationality. Therefore, our results showing that activating left DLPFC while inhibiting right DLPFC lead people to choose more future delayed loss may be essential to make people more “rational.”

For the negative events with social attributes, undergoing unpleasant experiences later (e.g., postponing vaccination to avoid pain from the needle prick or postponing schoolwork) leads to potential health risks and substantial societal costs. Our study did not include these types of intertemporal choice tasks. Future research should focus on these factors and test the effect of tDCS stimulation of DLPFC. Moreover, in a certain respect, because the bipolar tDCS montage may not be easy to disentangle for determining whether observed effects are caused by a specific hemisphere, future research should also explore behavioral changes by using stimulation targeted over a single region. For example, the experimental paradigm of bilateral stimulation, combined with two unilateral studies [A: anodal stimulation to left (F3+) DLPFC, right (F4+) DLPFC, and sham DLPFC stimulation; B: cathodal stimulations to left (F3−), right (F4−) DLPFC and sham stimulations], as suggested by Shen et al. (2016), can be adopted for intertemporal decision-making tasks related to loss. This will further clarify the activation and inhibition of the left or the right DLPFC separately.

Some cognitive mechanisms may have been involved in the effect of LH anodal/RH cathodal stimulation on intertemporal choice for loss. Studies have suggested that DLPFC is likely to play a vital role in impulse control and time perception (He et al., 2016). For example, Crews and Boettiger (2009) showed that the loss of DLPFC function is correlated with a lower level of inhibition control in the process of addictions. We considered the time perspective and measured future orientation after stimulation in our experiment. Future orientation, in which time is considered from the psychological perspective, refers to the orientation and processes through which individuals think about and plan for the future. The Consideration of Future Consequences scale introduced by Strathman et al. (1994) was used in our study. However, no significant treatment effect was noted (*F*_(2,56)_ = 1.124, *p* = 0.332). Neither LH anodal/RH cathodal tDCS nor RH anodal/LH cathodal tDCS showed a significant difference compared with sham stimulation. Therefore, time perception may not be a mechanism involved in the DLPFC’s role in intertemporal decision-making.

Furthermore, Shen et al. (2016) used high-definition tDCS to map changes in causal impulsivity through the bidirectional modulation of the DLPFC during intertemporal choice for gain, and anodal and cathodal stimulation of the left DLPFC decreased and increased impulsivity, respectively. Blain et al. (2016) also suggested that the impulsivity in intertemporal choices (i.e., the propensity to favor immediate rewards) increases under LPFC inhibition. Biological components underlying the empirical relationship between discounting and addiction may serve as criteria for a behavioral marker and elucidate the action mechanism of a disorder (Bickel et al., 2014). Therefore, future studies should further emphasize individuals’ inhibition control ability and its potential role in the effect of tDCS on intertemporal decision-making for both gains and losses. Additionally, tDCS stimulation of the DLPFC may have potential clinical applications for identifying the mechanism causing control loss in addiction (Noël et al., 2013).

Reducing physiological activity in the right DLPFC has been reported to lead to a preference for future larger gains compared with the sham condition (Cho et al., 2010). However, increasing evidence has revealed that the right DLPFC cannot affect the delay discounting rate (Hecht et al., 2013; He et al., 2016). In our study, although RH cathodal/LH anodal condition showed a similar trend of increase in future choice of loss as the LH anodal/RH cathodal condition, it did not reach statistical significance. Because of the large *p* values for the RH cathodal/LH anodal and sham conditions before and after stimulation, facilitating the right DLPFC while inhibiting the left DLPFC had a no significant effect on the intertemporal choice for loss. Steinbeis et al. (2012) presumed that the right DLPFC is normally involved in response inhibition instead of impulse control. We inferred that this may be related to the domain-specific effect in intertemporal choice. Intertemporal decision-making covers domains such as finance, health, addiction, and environment. König (2009) suggested that individuals have domain-specific effects on delay discounting. A study investigating US samples found that higher discounting rate of gain and lower discounting rate of loss were noted in the health domain, compared with the financial domain (Hardisty and Weber, 2009). We believed that this may be the reason that tDCS stimulation of the right DLPFC reduced cravings for sweet food but had no effect on monetary discounting tasks (Kekic et al., 2014). Future studies should adopt different domains of intertemporal choice tasks in tDCS studies to examine the role of the right DLPFC.

Moreover, the results of our study were not opposite between left anodal/right cathodal tDCS and right anodal/left cathodal tDCS. Several studies have explored hemispheric asymmetry (e.g., Davidson and Fox, 1989). fMRI studies have found evidence of hemispheric asymmetry in the human lateral prefrontal cortex during cognitive set-shifting (Konishi et al., 2002; Lie et al., 2006). DLPFC’s significant hemispheric asymmetry was also noted. Derrfuss et al. (2005) confirmed the hemispheric asymmetry of DLPFC during cognitive tasks using fMRI. Ko et al. (2008) revealed that impairing Montreal Card-Sorting task performance appeared to be limited only to the left DLPFC, whereas right DLPFC simulation did not have effects on the task performance. In addition to cognitive abilities, the hemispheric asymmetry of implicit temporal processes was also observed in Vallesi et al. (2006). Boggio et al. (2009) noted that bilateral stimulation can affect a larger cortical network and may result in a larger difference than a unilateral stimulation. Bifrontal direct current stimulation on DLPFC showed hemispheric asymmetry during risky-choice tasks (Ye et al., 2015) and delay discounting task with gains (Hecht et al., 2013). Therefore, our results support and confirm the hemispheric asymmetry in the field of intertemporal choice for loss. Because technologies develop rapidly, multimodal methods are starting to be used in neural science, such as tDCS-EEG (Mangia et al., 2014; Roy et al., 2014), indicating that we can simultaneously acquire electrophysiological data during high-definition tDCS by using high-resolution EEG. To further explore the real-time effect of tDCS on the hemispheric asymmetry of DLPFC, future studies may combine tDCS-EEG studies to obtain electrophysiological data during ongoing stimulation. Further exploration using tDCS-EEG techniques would improve our ability to target DLPFC for modulation and clinical rehabilitation procedures.

## Conclusion

Our experiment found that the bifrontal direct current stimulation can alter intertemporal decision-making of loss behavior in healthy individuals. Participants selected increasingly delayed options, instead of the immediate losses, when the left DLPFC was facilitated and the right DLPFC was inhibited. These preliminary findings contribute to the accumulating evidence that using noninvasive Transcranial Magnetic Stimulation and tDCS techniques in human studies have begun to reveal the causal link between frontal cortex activity and intertemporal decision-making (Essex et al., 2012; Kekic et al., 2014). Future studies may combine neuromodulation with neuroimaging measures to explore the neural changes, which may be applied to achieve utility maximization in neuroeconomics and have therapeutic implications on the treatment of addiction and procrastination.

## Ethics Statement

The participants were provided with informed written consent before entering the study, which was approved by the ethics committee of South China Normal University.

## Author Contributions

GX, XL, JH and QL participated in the design of this study, carried out the study and they all performed the statistical analysis. GX, XL, ZD and SC constructed the overall framework of the study and modified and polished it. GX and XL collected important background information and drafted the manuscript. All authors read and approved the final manuscript.

## Conflict of Interest Statement

The authors declare that the research was conducted in the absence of any commercial or financial relationships that could be construed as a potential conflict of interest.
